# Isolation of potent SARS-CoV-2 neutralizing antibodies and protection from disease in a small animal model

**DOI:** 10.1126/science.abc7520

**Published:** 2020-06-15

**Authors:** Thomas F. Rogers, Fangzhu Zhao, Deli Huang, Nathan Beutler, Alison Burns, Wan-ting He, Oliver Limbo, Chloe Smith, Ge Song, Jordan Woehl, Linlin Yang, Robert K. Abbott, Sean Callaghan, Elijah Garcia, Jonathan Hurtado, Mara Parren, Linghang Peng, Sydney Ramirez, James Ricketts, Michael J. Ricciardi, Stephen A. Rawlings, Nicholas C. Wu, Meng Yuan, Davey M. Smith, David Nemazee, John R. Teijaro, James E. Voss, Ian A. Wilson, Raiees Andrabi, Bryan Briney, Elise Landais, Devin Sok, Joseph G. Jardine, Dennis R. Burton

**Affiliations:** 1 Department of Immunology and Microbiology, The Scripps Research Institute, La Jolla, CA 92037, USA.; 2 Division of Infectious Diseases, Department of Medicine, University of California, San Diego, La Jolla, CA 92037, USA.; 3 IAVI Neutralizing Antibody Center, The Scripps Research Institute, La Jolla, CA 92037, USA.; 4 Consortium for HIV/AIDS Vaccine Development (CHAVD), The Scripps Research Institute, La Jolla, CA 92037, USA.; 5 IAVI, New York, NY 10004, USA.; 6 Center for Infectious Disease and Vaccine Research, La Jolla Institute for Immunology (LJI), La Jolla, CA 92037, USA.; 7 Center for Viral Systems Biology, The Scripps Research Institute, La Jolla, CA 92037, USA.; 8 Department of Pathology, George Washington University, Washington, DC 20052, USA.; 9 Department of Integrative Structural and Computational Biology, The Scripps Research Institute, La Jolla, CA 92037, USA.; 10 Ragon Institute of Massachusetts General Hospital, Massachusetts Institute of Technology, and Harvard University, Cambridge, MA 02139, USA.

## Abstract

Countermeasures to prevent and treat coronavirus disease 2019 (COVID-19) are a global health priority. We enrolled a cohort of severe acute respiratory syndrome coronavirus 2 (SARS-CoV-2)–recovered participants, developed neutralization assays to investigate antibody responses, adapted our high-throughput antibody generation pipeline to rapidly screen more than 1800 antibodies, and established an animal model to test protection. We isolated potent neutralizing antibodies (nAbs) to two epitopes on the receptor binding domain (RBD) and to distinct non-RBD epitopes on the spike (S) protein. As indicated by maintained weight and low lung viral titers in treated animals, the passive transfer of a nAb provides protection against disease in high-dose SARS-CoV-2 challenge in Syrian hamsters. The study suggests a role for nAbs in prophylaxis, and potentially therapy, of COVID-19. The nAbs also define protective epitopes to guide vaccine design.

## Protective neutralizing antibodies

Antibodies produced by survivors of coronavirus disease 2019 (COVID-19) may be leveraged to develop therapies. A first step is identifying neutralizing antibodies, which confer strong protection against severe acute respiratory syndrome coronavirus 2 (SARS-CoV-2). Rogers *et al.* used a high-throughput pipeline to isolate and characterize monoclonal antibodies from convalescent donors. Antibodies were selected for binding to the viral spike protein, which facilitates entry into host cells by binding to the angiotensin-converting enzyme 2 (ACE2) receptor. Most isolated antibodies bound to regions of the spike outside of the receptor binding domain (RBD); however, a larger proportion of the RBD-binding antibodies were neutralizing, with the most potent binding at a site that overlaps the ACE2 binding site. Two of the neutralizing antibodies were tested in Syrian hamsters and provided protection against SARS-CoV-2 infection.

*Science*, this issue p. 956

## Teaser

Passive transfer of a neutralizing antibody provides protection against disease in high-dose SARS-CoV-2 challenge in Syrian hamsters.

The novel coronavirus disease 2019 (COVID-19) has had devastating global health consequences, and there is currently no cure or licensed vaccine. Neutralizing antibodies (nAbs) to the causative agent of the disease, severe acute respiratory syndrome coronavirus 2 (SARS-CoV-2), represent potential prophylactic and therapeutic options and could help guide vaccine design. A nAb to another respiratory virus, respiratory syncytial virus (RSV), is in widespread clinical use prophylactically to protect vulnerable infants (*1*). Furthermore, nAbs prevent death from the emerging Ebola virus in macaques, even when given relatively late in infection, and thus have been proposed for use in outbreaks (*2*, *3*). Generally, nAbs with outstanding potency (known as super-antibodies) (*4*) can be isolated by deeply mining antibody responses of a sampling of infected donors. Outstanding potency coupled with engineering to extend antibody half-life from weeks to many months brings down the effective costs of antibodies and suggests more opportunities for prophylactic intervention. At the same time, outstanding potency can permit antiviral therapeutic efficacy that is not observed for less potent antibodies (*4*). Here, we present the isolation of highly potent nAbs to SARS-CoV-2 and demonstrate their in vivo protective efficacy in a small animal model, suggesting their potential utility as a medical countermeasure.

**Fig. 1:**
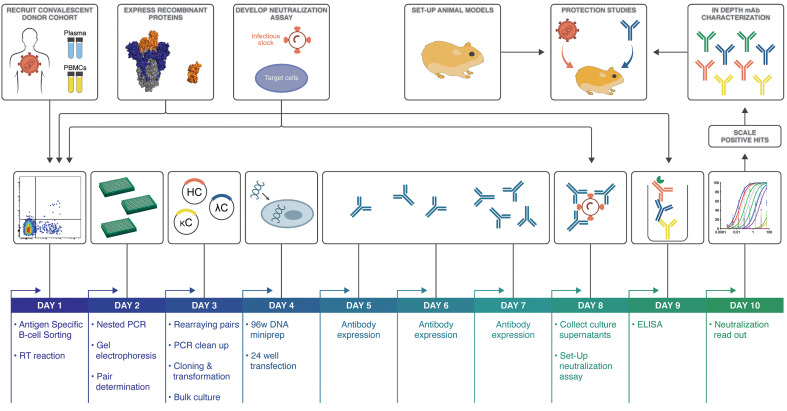
SARS-CoV-2 neutralizing antibody isolation strategy. A natural infection cohort was established to collect plasma and PBMC samples from individuals who recovered from COVID-19. In parallel, functional assays were developed to rapidly screen plasma samples for SARS-CoV-2 neutralizing activity. SARS-CoV-2 recombinant surface proteins were also produced for use as baits in single-memory B cell sorting and downstream functional characterization of isolated mAbs. Finally, a Syrian hamster animal model was set up to evaluate mAb passive immunization and protection. The standard mAb isolation pipeline was optimized to facilitate high-throughput amplification, cloning, expression, and functional screening of hundreds of unpurified Ab heavy and light chain pairs isolated from each of several selected neutralizers in only 10 days. Selected pairs were scaled up to purify IgG for validation and characterization experiments. Potent neutralizing mAbs were selected to evaluate protection in the Syrian hamster model. HC, heavy chain; κC, kappa light chain; ΛC, lambda light chain; RT, reverse transcriptase.

To investigate the antibody response against SARS-CoV-2 and discover nAbs, we adapted our pipeline to rapidly isolate and characterize monoclonal antibodies (mAbs) from convalescent donors (Fig. 1). A cohort of previously swab-positive SARS-CoV-2 donors was recruited for peripheral blood mononuclear cell (PBMC) and plasma collection. In parallel, we developed both live replicating and pseudovirus neutralization assays using a HeLa-ACE2 (angiotensin-converting enzyme 2) cell line that gave robust and reproducible virus titers. Convalescent serum responses were evaluated for neutralization activity against SARS-CoV-1 and SARS-CoV-2, and eight donors were selected for mAb discovery. Single antigen-specific memory B cells were sorted, and their corresponding variable genes were recovered and cloned using a high-throughput production system that enabled antibody expression and characterization in under 2 weeks. Promising mAbs were advanced for further biophysical characterization and in vivo testing.

## Development of viral neutralization assays

Two platforms were established to evaluate plasma neutralization activity against SARS-CoV-2, one using replication-competent virus and another using pseudovirus (PSV). Vero-E6 cells were first used as target cells for neutralization assays, but this system was relatively insensitive at detecting replicating virus compared with a HeLa cell line that stably expressed the cell surface ACE2 receptor (fig. S1A). The HeLa-ACE2 target cells gave reproducible titers and were used for the remainder of the study. In certain critical instances, HeLa-ACE2 and Vero cells were compared.

The live replicating virus assay used the Washington strain of SARS-CoV-2, USA-WA1/2020 (BEI Resources NR-52281) and was optimized to a 384-well format to measure plaque formation. In parallel, a PSV assay was established for both SARS-CoV-1 and SARS-CoV-2 using murine leukemia virus–based PSV (MLV-PSV) (*5*). The assay used single-cycle infectious viral particles bearing a firefly luciferase reporter for high-throughput screening. Unlike MLV-PSV, which buds at the plasma membrane, coronaviruses assemble in the endoplasmic reticulum (ER)–Golgi intermediate compartment, so the C terminus of the SARS-CoV-1 spike (S) protein contains an ER retrieval signal (*6*). The alignment of SARS-CoV-1 and SARS-CoV-2 S proteins showed that this ER retrieval signal is conserved in SARS-CoV-2 (fig. S1B). To prepare high titers of infectious SARS-CoV-1 and SARS-CoV-2 PSV particles, various truncations of SARS-CoV-1 and SARS-CoV-2 S protein were expressed in which the ER retrieval signal was removed to improve exocytosis of the virus. Pseudovirion versions carrying SARS-CoV1-SΔ28 and SARS-CoV2-SΔ18S protein efficiently transduced ACE2-expressing target cells but not control HeLa or A549 cells (fig. S1C). Control VSV-G pseudotyped virions showed a similar transduction efficiency in all target cells. Luciferase expression in transduced cells proved to be proportional to viral titer over a wide range (fig. S1D).

## Establishment of a SARS-CoV-2 cohort

**Fig. 2:**
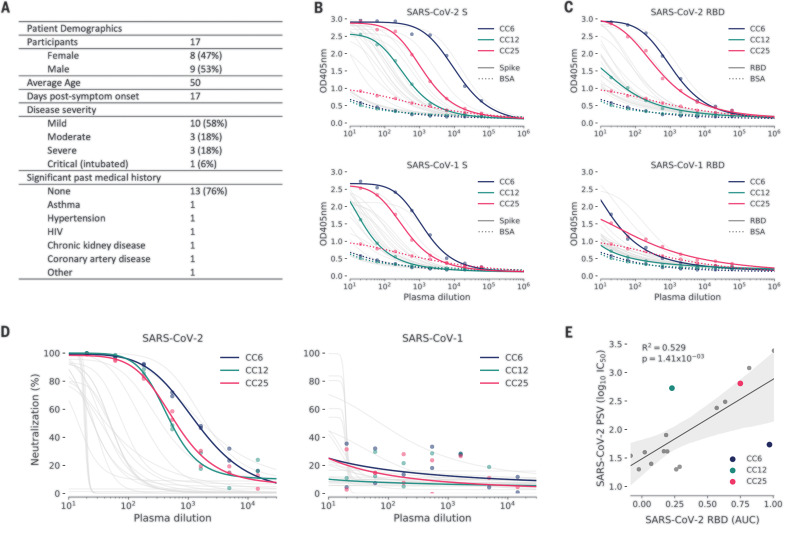
COVID-19 cohort functional screening. (**A**) Demographics of the University of California, San Diego (UCSD) COVID-19 cohort (CC) participants. CC plasma was tested for binding to SARS-CoV-1 and SARS-CoV-2 S proteins (**B**) and RBD subunits (**C**) by ELISA. Background binding of plasma to bovine serum albumin–coated plates is represented by a dotted line. OD405nm, optical density for wavelength of 405 nm. (**D**) Plasma was also tested for neutralization of pseudotyped (PSV) SARS-CoV-1 and SARS-CoV-2 virions. (**E**) Correlation between PSV SARS-CoV-2 neutralization and RBD subunit ELISA binding AUC (area under the curve). AUC was computed using Simpson’s rule. The 95% confidence interval of the regression line is shown by the gray shaded area and was estimated by performing 1000 bootstrap resamplings. *R*^2^ (coefficient of determination) and *P* values of the regression are also indicated. CC participants from whom mAbs were isolated are specifically highlighted in blue (CC6), green (CC12), and pink (CC25).

In parallel to the development of neutralization assays, a cohort was established in San Diego, California, of 17 donors who had previously been infected with SARS-CoV-2 (Fig. 2A, fig. S2A, and table S1). The cohort was 47% female, and the average age was 50 years. Infection was determined by a positive SARS-CoV-2 polymerase chain reaction (PCR) test from a nasopharyngeal swab. All donors also had symptoms consistent with COVID-19, and disease severity ranged from mild to severe, including intubation in one case, although all donors recovered. Donor plasma were tested for binding to recombinant SARS-CoV-2 and SARS-CoV-1 S and receptor binding domain (RBD) proteins, for binding to cell surface–expressed spikes, and for neutralization in both live replicating virus and PSV assays [Fig. 2, B to D, and fig. S2B; three donors (CC6, CC12, and CC25) that are further discussed below are highlighted]. Binding titers to SARS-CoV-2 S protein varied considerably, reaching a half-maximal effective concentration (EC_50_) at serum dilutions of ~10^4^, with titers against the RBD about an order of magnitude less. Titers against SARS-CoV-1 S protein were notably less than those for SARS-CoV-2 S protein, and titers against SARS-CoV-1 RBD were only detected in a small number of donors. Neutralizing titers in the PSV assay varied over a wide range for SARS-CoV-2 (Fig. 2D and fig. S2A) and were low or undetectable against SARS-CoV-1. RBD binding and PSV neutralization were notably well correlated (Fig. 2E). There was also a positive correlation between cell surface spike binding and live replicating virus neutralization (fig. S2C). The titers in the PSV assay and the replicating virus assay were largely similar (figs. S2 and S3). In most later measurements, the PSV assay was preferred owing to its higher throughput.

## Antibody isolation and preliminary functional screens for down-selection

Cryopreserved PBMCs from eight donors were stained for memory B cells markers (CD19^+^/IgG^+^; IgG, immunoglobulin G) and both AviTag biotinylated RBD and SARS-CoV-2 S antigen baits before single-cell sorting. S^+^ and S^+^/RBD^+^ memory B cells were present at an average frequency of 2.0 and 0.36%, respectively, across the eight donors (fig. S4A). In total, 3160 antigen-positive (Ag^+^) memory B cells were sorted to rescue native heavy and light chain pairs for mAb production and validation (fig. S4B). A total of 2045 antibodies were cloned and expressed, which represents, on average, a 65% PCR recovery of paired variable genes and >86% estimated recovery of fully functional cloned genes (fig. S4C). The bulk-transformed ligation products for both the heavy chain and light chain were transfected and tested for binding to RBD and S protein and for neutralization in the SARS-CoV-2 PSV assay using HeLa-ACE2 target cells (fig. S5).

**Fig. 3:**
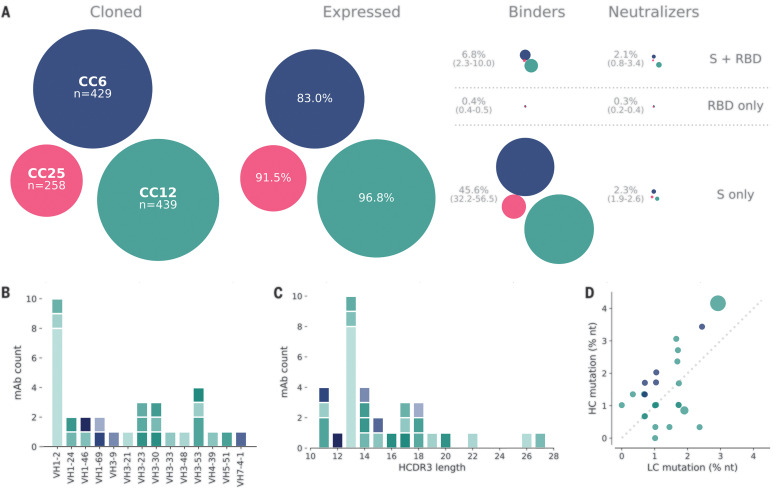
Antibody isolation and functional screening for SARS-CoV antigen binding and neutralization. (**A**) Antibody down-selection process from three donors, presented as bubble plots. The areas of the bubbles for each donor are sized according to the number of antibodies (n) that were cloned and transfected, then scaled according to the number that were positive in subsequent assays. All antibodies that expressed at measurable levels were tested for binding to S protein and RBD to determine their specificity and then screened for neutralization. (**B**) VH gene distribution of down-selected mAbs. (**C**) Heavy chain CDR3 lengths of down-selected mAbs. Antibodies in (B) and (C) are colored according to their respective clonal lineages. (**D**) Mutation frequency of down-selected mAb lineages. Bubble position represents the mean mutation frequency for each lineage, with a bubble area that is proportional to lineage size. LC, light chain; nt, nucleotides.

The majority of transfected pairs (92%) resulted in IgG expression. Of these, 43% showed binding only to S protein, while 5.9% bound to both S and RBD proteins and 0.1% bound only to RBD. The supernatants were also screened for binding to an unrelated HIV antigen (BG505 SOSIP) to eliminate nonspecific or polyreactive supernatants. The supernatants were next evaluated for neutralization activity using SARS-CoV-2 and SARS-CoV-1 pseudoviruses. A small proportion of the binding antibodies showed neutralization activity, and, unexpectedly, that activity was equally distributed between RBD^+^/S^+^ and S^+^-only binders, despite a much larger number of S^+^-only binding supernatants, as exemplified by the three donors, CC6, CC12, and CC25 (Fig. 3A). These data indicate that viral infection generates a strong response against the non-RBD regions of the S protein, but only a small proportion of that response is neutralizing. In contrast, there are fewer RBD-binding antibodies, but a larger proportion of these neutralize SARS-CoV-2 pseudovirus. Antibodies that tested positive for neutralization in the high-throughput screening were sequence-confirmed and advanced for large-scale expression for additional characterization.

Thirty-three antibodies were prioritized for in-depth characterization from the three donors, CC6, CC12, and CC25. Within that subset, we identified 25 distinct lineages, with 23 containing a single member (table S2). VH1 and VH3 gene families were notably prominent in these Abs, and there was a diversity of CDR3 lengths (Fig. 3, B and C). There was one prominent example of a clonally expanded lineage, with eight recovered clonal members that averaged 4.3 and 2.8% mutations from germline at the nucleotide level in the heavy chain and light chain, respectively (Fig. 3D). The remaining clones were relatively unmutated, averaging just above 1% mutation at the nucleotide level, suggesting that these antibodies were primed by the ongoing COVID infection and likely not recalled from a previous endemic human coronavirus exposure. All antibodies that were expressed at scale were evaluated in standard enzyme-linked immunosorbent assay (ELISA)–based polyreactivity assays with solubilized Chinese hamster ovary (CHO) membrane preparations, single-stranded DNA, and insulin (*7*, *8*), and none were polyreactive (fig. S6).

## Functional activity of down-selected antibodies

**Fig. 4:**
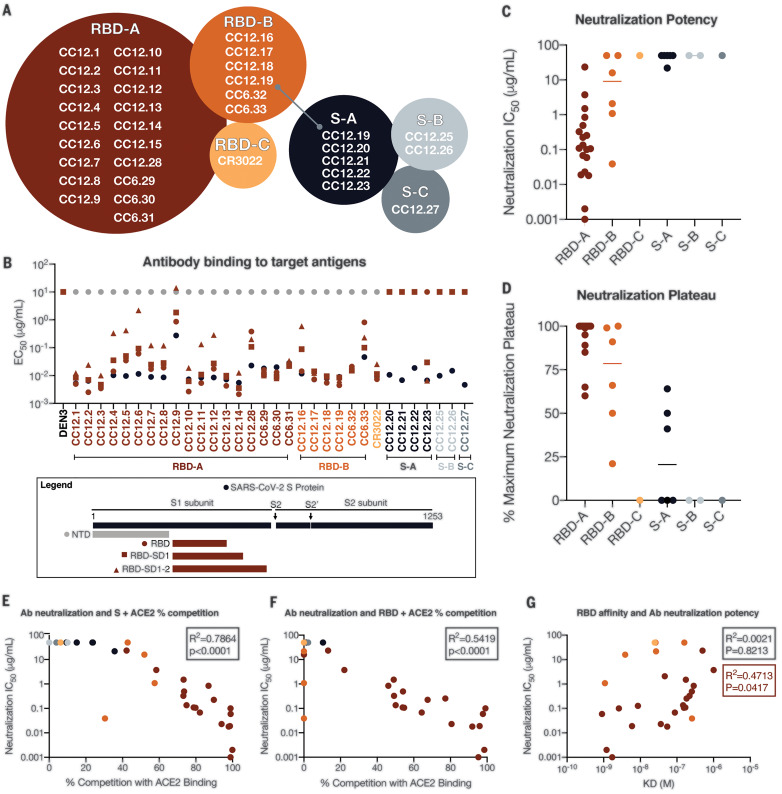
Antibody functional activity by epitope specificities. Monoclonal antibody epitope binning was completed using RBD and SARS-CoV-2 S protein as target antigens. (**A**) A total of three noncompeting epitopes for RBD (RBD-A, RBD-B, and RBD-C) and three noncompeting epitopes for S (S-A, S-B, and S-C) were identified. (**B**) MAbs were evaluated for binding to different target antigens (S, NTD, RBD, RBD-SD1, and RBD-SD1-2) by ELISA and apparent EC_50_ values are reported in micrograms per milliliter. (**C**) MAbs were evaluated for neutralization of SARS-CoV-2 pseudovirus using HeLa-ACE2 target cells. Antibodies are grouped according to epitope specificities, and neutralization IC_50_ values are reported in micrograms per milliliter. (**D**) The MNP is reported for each mAb and grouped by epitope specificity. MAbs were mixed with (**E**) S or (**F**) RBD protein and measured for binding to HeLa-ACE2 target cells as a measure of competition to the cell surface ACE-2 receptor. (**G**) mAb neutralization potencies (IC_50_) are plotted as a function of dissociation constants (KD) measured by SPR to RBD target antigen.

The antibody hits that were identified in the high-throughput screening were next evaluated for epitope specificity by biolayer interferometry using S and RBD proteins as capture antigens. The antigens were captured on anti-HIS biosensors before addition of saturating concentrations (100 μg/ml) of antibodies that were then followed by competing antibodies at a lower concentration (25 μg/ml). Accordingly, only antibodies that bind to a noncompeting site would be detected in the assay. Among the antibodies evaluated, the results reveal three epitope bins for RBD (designated RBD-A, RBD-B, and RBD-C) and three epitope bins for the S protein (designated S-A, S-B, and S-C) (Fig. 4A and fig. S7). The mAb CC12.19 appears to compete with antibodies targeting two different epitopes, RBD-B and S-A (fig. S7), which might indicate that this mAb targets an epitope spanning RBD-B and S-A. To evaluate epitope specificities further, we next assessed binding of the antibodies to extended RBD constructs with subdomains (SD) 1 and 2, including the independently folding RBD-SD1 and RBD-SD1-2 and the N-terminal domain (NTD) (Fig. 4B and fig. S8, A and B). None of the antibodies showed binding to the NTD. CC12.19 binds to all other constructs, which supports the epitope binning data described in Fig. 4A. The other antibodies grouped in the S-A epitope bin that compete with CC12.19 either showed no binding to RBD or RBD-SD constructs (CC12.20 and CC12.21) or showed binding to RBD-SD1 and RBD-SD1-2 but not RBD (CC12.23). These data suggest two competing epitopes within the S-A epitope bin: one that is confined to the non-RBD region of the S protein, and one that includes some element of RBD-SD1-2. This interpretation will require further investigation by structural studies.

We next evaluated the mAbs for neutralization activity against SARS-CoV-2 and SARS-CoV-1 pseudoviruses. The neutralization half-maximal inhibitory concentration (IC_50_) potencies of these antibodies are shown in Fig. 4C, and their associated maximum neutralization plateaus (MNPs) are shown in Fig. 4D. A comparison of neutralization potencies between pseudovirus (fig. S8C) and live replicating virus (fig. S8D) is also included. The most potent neutralizing antibodies were those directed to RBD-A epitope, including two antibodies, CC6.29 and CC6.30, that neutralize SARS-CoV-2 pseudovirus with an IC_50_ of 2 ng/ml and 1 ng/ml, respectively (Fig. 4C). In comparison, antibodies directed to RBD-B tended to have a higher IC_50_ and many plateaued below 100% neutralization. Despite this trend, CC6.33 is directed against RBD-B and showed complete neutralization of SARS-CoV-2 with an IC_50_ of 39 ng/ml and also neutralized SARS-CoV-1 with an IC_50_ of 162 ng/ml. CC6.33 was the only antibody that showed potent neutralization of both pseudoviruses. The antibodies that do not bind to RBD and are directed to non-RBD epitopes on the S protein all showed poor neutralization potencies and MNPs well below 100%.

To evaluate whether the RBD-A epitope might span the ACE2 binding site, we next performed cell surface competition experiments. Antibodies were premixed with biotinylated S (Fig. 4E) or RBD (Fig. 4F) proteins at a 4:1 molar ratio of antibodies to target antigen. The mixture was then incubated with the HeLa-ACE2 cell line and the percent competition against ACE2 receptor was recorded by comparing percent binding of the target antigen with and without antibody present (fig. S8E). The antibodies targeting the RBD-A epitope competed best against the ACE2 receptor, and the neutralization IC_50_ correlated well with the percent competition for ACE2 receptor binding for both S protein (Fig. 4E) and RBD (Fig. 4F). We also assessed the affinity of all RBD-specific antibodies to soluble RBD by surface plasmon resonance (SPR) and found a poor correlation between affinity and neutralization potency (Fig. 4G and fig. S9). However, the correlation is higher when limited to antibodies targeting the RBD-A epitope. The lack of a correlation between RBD binding and neutralization for mAbs contrasts with the strong correlation described earlier for serum RBD binding and neutralization. Overall, the data highlight epitope RBD-A as the preferred target for eliciting neutralizing antibodies and suggest that corresponding increases in affinity of mAbs to RBD-A will likely result in corresponding increases in neutralization potency.

SARS-CoV-2 has shown some propensity for mutation as it has circulated worldwide, as evidenced, for example, in the emergence of the D614G variant (*9*). We investigated the activity of five nAbs against six viral variants that have been reported. The three sera studied above (CC6, CC12, and CC25) neutralized all the variants (fig. S10A). All five nAbs neutralized the D614G variant. However, one variant with a mutation in the ACE2 binding site [Gly^476^→Ser (G476S)] did show effectively complete resistance to one of the nAbs, and another variant (V367F) showed a 10-fold higher IC_50_ than the WA1 strain (fig. S10B).

## Passive transfer of neutralizing antibodies and SARS-CoV-2 challenge in Syrian hamsters

**Fig. 5:**
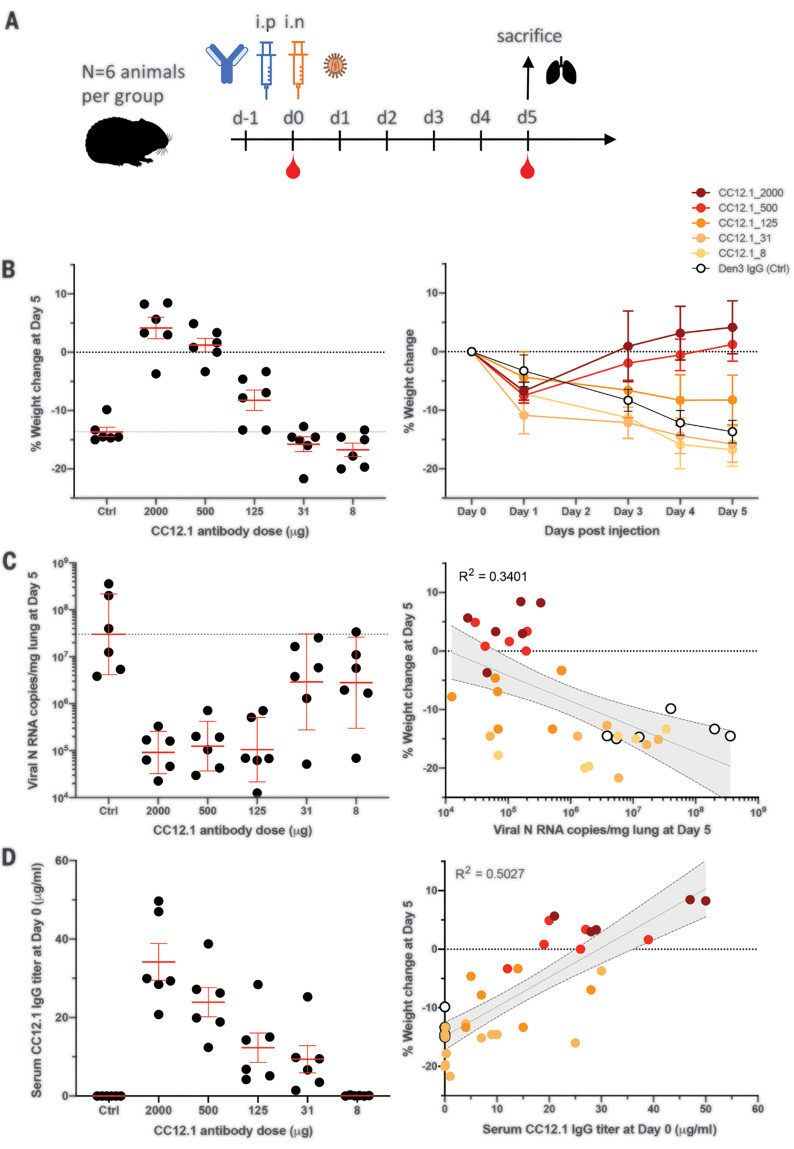
A potent SARS-CoV-2 RBD-specific neutralizing mAb protects against weight loss and lung viral replication in Syrian hamsters. (**A**) SARS-CoV-2–specific human neutralizing mAb CC12.1 isolated from natural infection was administered at a starting dose of 2 mg per animal (average: 16.5 mg/kg) and subsequent serial fourfold dilutions. Control animals received 2 mg of Den3. Each group of six animals was challenged intranasally (i.n.) 12 hours after infusion with 1 × 10^6^ PFU of SARS-CoV-2. Serum was collected at the time of challenge (day 0), and animal weight was monitored as an indicator of disease progression. On day 5, lung tissue was collected for viral burden assessment. (**B**) Percent weight change was calculated from day 0 for all animals. (**C**) Viral load, as assessed by nucleocapsid RNA quantitative polymerase chain reaction (qPCR) from lung tissue at day 5 after infection. (**D**) Serum titers of the passively administered mAb, as assessed by ELISA at the time of challenge [12 hours after intraperitoneal (i.p.) administration]. Correlation analyses with 95% confidence intervals indicated by the gray shaded area.

To investigate the relationship between in vitro neutralization and protection in vivo against SARS-CoV-2, we selected two mAbs for passive transfer and challenge experiments in a Syrian hamster animal model on the basis of a summary of the nAb data (table S3 and fig. S11). The experimental design for the passive transfer study is shown in Fig. 5A. In the first experiment, we tested nAb CC12.1, which targets the RBD-A epitope and has an in vitro IC_50_ neutralization of 0.019 μg/ml against pseudovirus, and in the second experiment, we tested nAb C12.23, which targets the S-B epitope with an IC_50_ neutralization of 22 μg/ml. In both experiments, an unrelated antibody to dengue virus, Den3, was used as a control. The anti-SARS-CoV-2 nAbs were delivered at five different concentrations to evaluate dose-dependent protection, starting at 2 mg per animal (average: 16.5 mg/kg) at the highest dose and 8 μg per animal at the lowest dose. The Den3 control antibody was delivered at a single dose of 2 mg per animal. Sera were collected from each animal 12 hours after intraperitoneal infusion of the antibody, and all animals were subsequently challenged with a dose of 1 × 10^6^ plaque forming units (PFU) of SARS-CoV-2 (USA-WA1/2020) by intranasal administration 12 hours after antibody infusion (Fig. 5A).

Syrian hamsters typically clear virus within 1 week after SARS-CoV-1 infection, and observations made in that model system determined the strategy adopted here (*10*). The hamsters were weighed daily as a measure of disease due to infection. Lung tissues were collected to measure viral load on day 5. A data summary is presented in Fig. 5B and fig. S12A for animals that received CC12.1, which targets the RBD-A epitope. The control animals that received Den3 lost, on average, 13.6% of body weight by day 5 after virus challenge. In comparison, the animals that received the neutralizing RBD-A antibody at a dose of 2 mg (average: 16.5 mg/kg) or 500 μg (average: 4.2 mg/kg) exhibited no weight loss. However, animals that received a dose of 125 μg (average: 0.9 mg/kg) had an average body weight loss of 8%, while animals that received a dose of 31 μg/ml (average: 0.2 mg/kg) and 8 μg/ml (average: 0.06 mg/kg) lost 15.8 and 16.7% of body weight, respectively. Although these animals showed a trend for greater weight loss than did control animals, this trend did not achieve statistical significance (table S4). Given concerns about antibody-mediated enhanced disease in SARS-CoV-2 infection, this observation merits further attention using larger animal group sizes. The weight loss data are further corroborated by quantification of lung viral load measured by real-time PCR (Fig. 5C), which showed a moderate correlation to weight loss. The data indicate comparable viral loads between the three higher doses (2 mg, 500 μg, and 125 μg) of nAbs. In contrast, equivalent viral loads were observed between the control group receiving Den3 and the low-dose groups receiving 31 or 8 μg of nAb. In contrast to the nAb to RBD-A, the less potent and incompletely neutralizing antibody to the S-B epitope showed no evidence of protection at any concentration when compared with control animals (fig. S12B).

To determine the antibody serum concentrations that may be required for protection against disease from SARS-CoV-2 infection, we also measured the antibody serum concentrations just before intranasal virus challenge (Fig. 5D). The data highlight that an antibody serum concentration of ~22 μg/ml of nAb (1160 × PSV neutralization IC_50_) enables full protection and a serum concentration of 12 μg/ml (630 × PSV neutralization IC_50_) is adequate for a 50% reduction in disease, as measured by weight loss. The effective antibody concentration required at the site of infection to protect from disease remains to be determined. Sterilizing immunity at serum concentrations that represent a large multiplier of the in vitro neutralizing IC_50_ is observed for many viruses (*11*).

## Discussion

Using a high-throughput rapid system for antibody discovery, we isolated more than 1000 mAbs from three convalescent donors by memory B cell selection using SARS-CoV-2 S or RBD recombinant proteins. About half of the mAbs isolated could be expressed, and they also bound effectively to S and/or RBD proteins. Only a small fraction of these Abs were neutralizing, which highlights the value of deep mining of responses to access the most potent Abs (*4*).

A range of nAbs were isolated to different sites on the S protein. The most potent Abs, reaching single-digit nanogram per milliliter IC_50_ values in PSV assays, are targeted to a site that, judged by competition studies, overlaps the ACE2 binding site. Only one of the Abs, directed to RBD-B, neutralized SARS-CoV-1 PSV, as may be anticipated given the differences in ACE2 contact residues between the two viruses (fig. S13) and given that the selections were performed with SARS-CoV-2 target proteins. Abs that are directed to the RBD but not competitive with soluble ACE2 (although they may be competitive in terms of an array of membrane-bound ACE2 molecules interacting with an array of S proteins on a virion) are generally less potent neutralizers and tend to show incomplete neutralization, plateauing well below 100% neutralization. The one exception is the cross-reactive RBD-B antibody, mentioned above. Similarly low potency and incomplete neutralization are observed for Abs to the S protein that are not reactive with recombinant RBD. The cause(s) of these incomplete neutralization phenomena is unclear but presumably originates in some S protein heterogeneity that is either glycan, cleavage, or conformationally based. Regardless, the RBD-A nAbs that directly compete with ACE2 are clearly the most preferred for prophylactic and therapeutic applications and as reagents to define nAb epitopes for vaccine design. Note that, even for a small sampling of naturally occurring viral variants, two were identified that showed notable resistance to individual potent nAbs to the WA1 strain, which suggests that neutralization resistance will need to be considered in planning for clinical applications of nAbs. Cocktails of nAbs may be required.

In terms of nAbs as passive reagents, the efficacy of a potent anti-RBD nAb in vivo in Syrian hamsters is promising in view of the positive attributes of this animal model (*12*) and suggests that human studies are merited. Nevertheless, as for any animal model, there are many limitations, including, in the context of antibody protection, differences in effector cells and Fc receptors between humans and hamsters. The failure of the non-RBD S-protein nAb to protect in the animal model is consistent with its lower potency and, likely most importantly, its inability to fully neutralize challenge virus. In the context of human studies, the following antibody engineering goals could be considered: improving the potency of protective nAbs by enhancing binding affinity to the identified RBD epitope, improving half-life, and reducing Fc receptor binding to minimize potential antibody-dependent enhancement (ADE) effects if they are identified. As observed for heterologous B cell responses against different serotypes of flavivirus infection, there is a possibility, but no current experimental evidence, that subtherapeutic vaccine serum responses or subtherapeutic nAb titers could potentially exacerbate future coronavirus infection disease burden by expanding the viral replication and/or cell tropism of the virus. If ADE is found for SARS-CoV-2 and operates at subneutralizing concentrations of neutralizing antibodies, as it can for dengue virus (*13*), then it would be important, from a vaccine standpoint, to carefully define the full range of nAb epitopes on the S protein, as we have begun to do here. From a passive antibody standpoint, it would be important to maintain high nAb concentrations or appropriately engineer nAbs.

The nAbs described here have very low somatic hypermutation (SHM), typically only one or two mutations in the VH gene and one or two in the VL gene. Such low SHM may be associated with the isolation of the nAbs relatively soon after infection, perhaps before affinity-maturation has progressed. Low SHM has also been described for potent nAbs to Ebola virus, RSV, Middle East respiratory syndrome coronavirus, and yellow fever virus (*14*–*17*) and may indicate that the human naïve repertoire is often sufficiently diverse to respond effectively to many pathogens with little mutation. Of course, nAb efficacy and titer may increase over time, as described for other viruses, and it will be interesting to see if even more potent nAbs to SARS-CoV-2 evolve in our donors in the future.

What do our results suggest for SARS-CoV-2 vaccine design? First, they suggest a focus on the RBD—strong nAb responses have indeed been demonstrated by immunizing mice with a multivalent presentation of RBD (*18*). The strong preponderance of non-neutralizing antibodies and the very few nAbs to S protein that we isolated could arise for a number of reasons, including the following: (i) The recombinant S protein that we used to select B cells is a poor representation of the native spike on virions. In other words, there may be many nAbs to the S protein, but we failed to isolate them because of the selecting antigen. (ii) The recombinant S protein that we used is close to native, but non-neutralizing antibodies bind to sites on the S protein that do not interfere with viral entry. (iii) The S protein in natural infection disassembles readily, generating a strong Ab response to viral debris that is non-neutralizing, because the antibodies recognize protein surfaces that are not exposed on the native spike. The availability of both neutralizing and non-neutralizing antibodies generated in this study will facilitate evaluation of S protein immunogens for presentation of neutralizing and non-neutralizing epitopes and will promote effective vaccine design. The design of an immunogen that improves on the quality of nAbs elicited by natural infection may well emerge as an important goal of vaccine efforts (*19*).

In summary, we describe the very rapid generation of neutralizing antibodies to a newly emerged pathogen. The antibodies can find clinical application and will aid in vaccine design.

## Supplementary Materials

science.sciencemag.org/content/369/6506/956/suppl/DC1 Materials and Methods Figs. S1 to S13 Tables S1 to S4 References (*20*–*27*) MDAR Reproducibility Checklist

## Appendix

View/request a protocol for this paper from *Bio-protocol*.
